# Single-incision assisted laparoscopic surgery (SILS) in the treatment of an intussusception induced by a solitary hamartomatous polyp: a case report and review of the literature

**DOI:** 10.1186/s13256-015-0606-8

**Published:** 2015-06-02

**Authors:** Michael Pitiakoudis, Konstantinos Romanidis, Alexandra Giatromanolaki, Nikos Courcoutsakis, Eleni-Aikaterini Nagorni, Soultana Foutzitzi, Alexandra Tsaroucha, Petros Zezos, Georgios Kouklakis

**Affiliations:** 1 Second Department of Surgery, Democritus University of Thrace, University Hospital of Alexandroupolis, Dragana, 68100 Alexandroupolis Greece; 2 Department of Pathology, University General Hospital of Alexandroupolis, Dragana, 68100 Alexandroupolis Greece; 3 Department of Radiology and Medical Imaging, University Hospital of Alexandroupolis, Dragana, 68100 Alexandroupolis Greece; 4 Gastrointestinal Endoscopy Unit, Democritus University of Thrace, University General Hospital of Alexandroupolis, Dragana, 68100 Alexandroupolis Greece

**Keywords:** Bowel intussusception, Single-incision laparoscopic surgery, Solitary Peutz-Jeghers-type polyp

## Abstract

**Introduction:**

In this case report, we describe the successful treatment of a small-bowel intussusception, which was caused by a 3cm solitary hamartomatous polyp, with single-incision laparoscopic surgery. Single-incision laparoscopic surgery is a minimally invasive surgical procedure with important advantages that allows the reduction of the intussusception and the resection of the polyp. This case report contributes to the medical literature by describing the advantages of this surgical technique that warrant its consideration as a treatment of choice in similar cases.

**Case presentation:**

We report a case of a 19-year-old Greek woman who complained about intermittent, non-specific abdominal pain in her left lateral abdomen. She had been admitted to the hospital because of incomplete obstructive ileus. Ultrasound and computed tomography were carried out, which revealed an intussusception of the small bowel. This pathogenic situation was treated by single-incision laparoscopic surgery. Her pathology report revealed a benign, hamartomatous excised polyp of the Peutz-Jeghers type. The patient had a quick recovery without any post-operative complications.

**Conclusion:**

We recommend single-incision laparoscopic surgery for the safe excision of solitary hamartomatous polyps and the management of their complications, as it represents a potential advance in minimally invasive approaches.

## Introduction

Hamartomatous bowel polyps can occur anywhere in the gastrointestinal tract and can grow large enough to cause bowel obstructions in combination with their pedunculated nature [1-5]. Usually broad-based and multiple, polyps vary in size from a few millimeters to several centimeters in diameter [2]. The presenting symptoms include abdominal pain (23%), rectal bleeding (14%), anemia, nausea and vomiting. This pathological type of polyp usually accompanies the Peutz-Jeghers syndrome (PJS) [1,6,7]. PJS is an autosomal dominant inherited disease with an incidence of 1 in 150,000. It is characterized by hamartomatous polyps, which are usually multiple, throughout the length of the gastrointestinal tract and by melanin deposits in the lips, buccal mucosa, peri-oral area and facial skin. PJS arises in the second and third decades of life [1,6]. The male-to-female ratio is 1:1 [3]. Gastrointestinal obstruction, rectal bleeding (14%) and perforation are some possible surgical complications, and especially obstructive ileus, that can be caused either directly by a sizable polyp or indirectly by intussusception (45%) [4,7]. Although the incidence of these acute complications is rare, as they depend on the size and location of the polyp, the surgical approach should be aggressive and effective as well [3]. In this report, we present a case of acute small bowel intussusception that was caused by a 3cm solitary polyp, which was managed by performing single-incision laparoscopic surgery (SILS), and we describe the post-operative pathological characteristics of Peutz-Jeghers polyp (PJP).

## Case presentation

**Figure 1:**
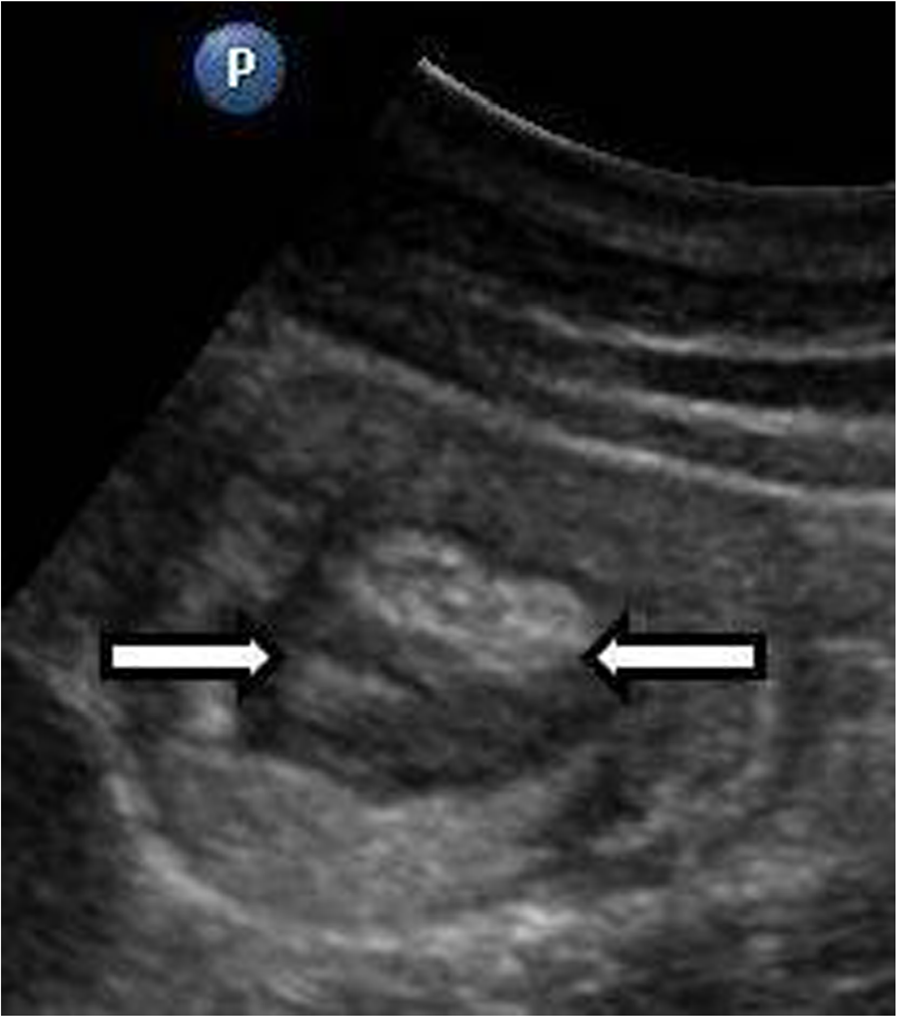
Abdominal ultrasonogram. Small bowel loop with thickened wall and round lesion within the intestinal lumen indicates polyp (arrows).

**Figure 2:**
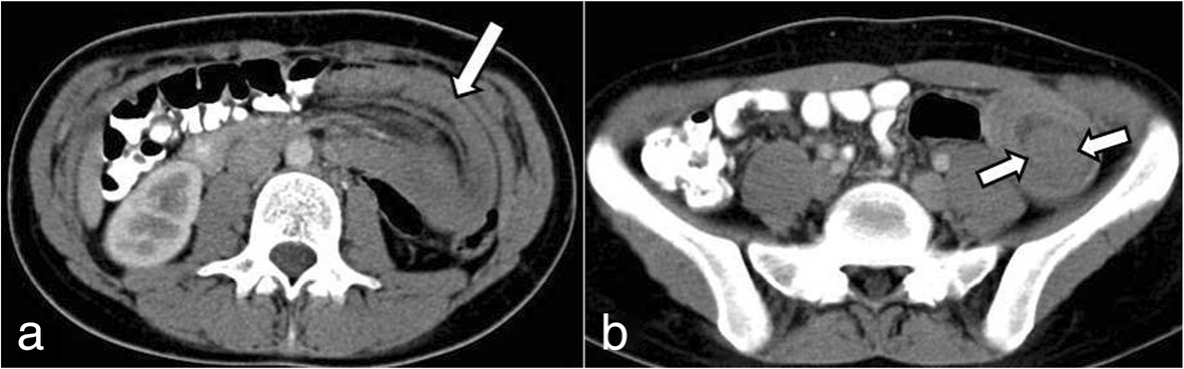
Abdominal computed tomography. **(a)** Small bowel intussusception (arrow). **(b)** A polyp within the intussuscepted loop (arrows).

**Figure 3:**
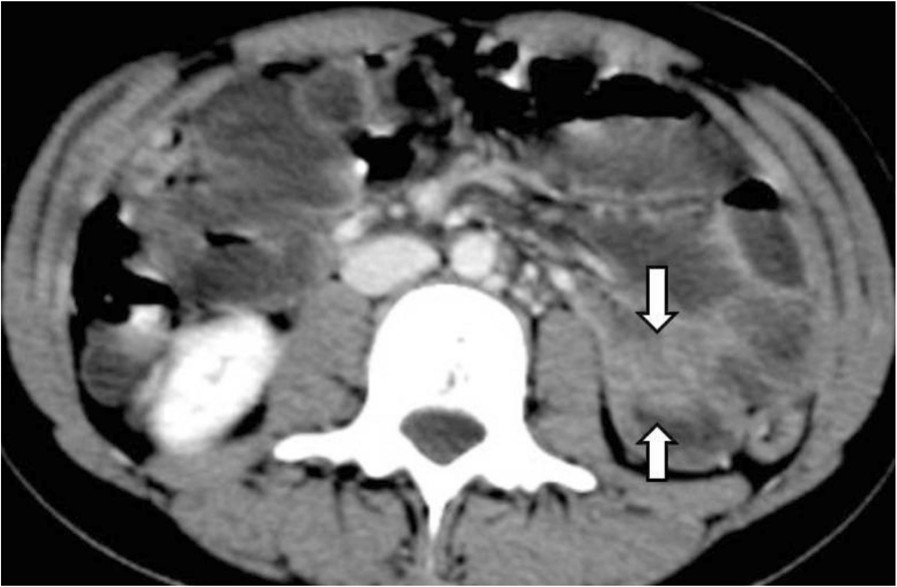
Computed tomographic enteroclysis. Neutral enteral contrast shows a polypoid intraluminal mass with remarkable enhancement (arrows).

A 19-year-old Greek woman consulted at the emergency department for recurrent episodes of non-specific cramping and intermittent abdominal pain in her left lateral abdomen of 4 days’ duration. The pain was accompanied by nausea and vomiting. She had already been admitted to the hospital two times in the past with symptoms of incomplete obstructive ileus accompanied by partial retention of gas and feces. The patient had no significant past medical history, reported no tobacco use or alcohol intoxication and was on no medications. Her family history was also not significant, and thus her symptoms were not deemed to be associated with inherited PJS. Her general condition and vital signs were normal. Her physical examination was unremarkable, except for a mild tenderness to the left lateral abdomen. On the day of admission, the results of her routine laboratory investigations were within normal ranges, except for mildly increased leukocytes, which were measured at 15,200/μl (normal range: 3,500 to 10,800/μl). Ultrasound (US) of the abdomen was performed, which revealed a target-like mass that suggested an intussusception of the small bowel to the left abdominal quadrant and very closely to the ligament of Treitz (Figure 1). Computed tomography (CT) was then immediately carried out to clarify the US findings. The findings were in agreement with the sausage-type appearance of intussusception visualized on the CT scan, with alternating areas of high and low attenuation (Figure 2). Continually, CT enteroclysis was carried out to scan the whole intestine for the existence of other polyps. However, this technique confirmed only the solitary polyp close to the ligament of Treitz (Figure 3).

**Figure 4:**
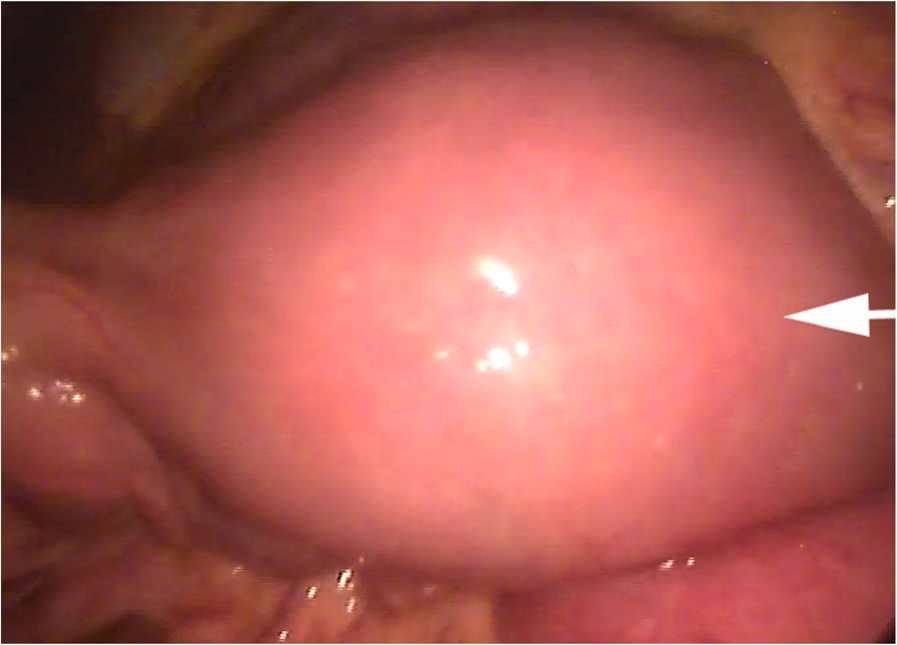
Intra-operative view of the bowel segment with the solitary polyp (arrow) after the intussusception reduction.

After a pre-operative consultation with the patient and her family, we performed SILS. The patient was placed in the Lloyd-Davies position. Afterward, we performed a 25mm incision to the patient’s umbilicus. We used SILS™ Port 5mm-12mm (Covidien™; Medtronic, Minneapolis, MN, USA), which we also used to create a pneumoperitoneum. By using the patient’s umbilicus as a single port, first we recognized the pathogenic segment of the small bowel at a distance of 90cm from the ligament of Treitz, and continually we managed to reduce the intussusception by using articulate graspers (Figure 4). Then, we exteriorized the polyp-bearing small intestine through the umbilicus port, resected the solitary polyp and excised the invalid part of the bowel by performing a side-to-side enterostomy using Endo GIA Universal Single-Use Stapler 12mm AutoSuture (Covidien™; Medtronic).

**Figure 5:**
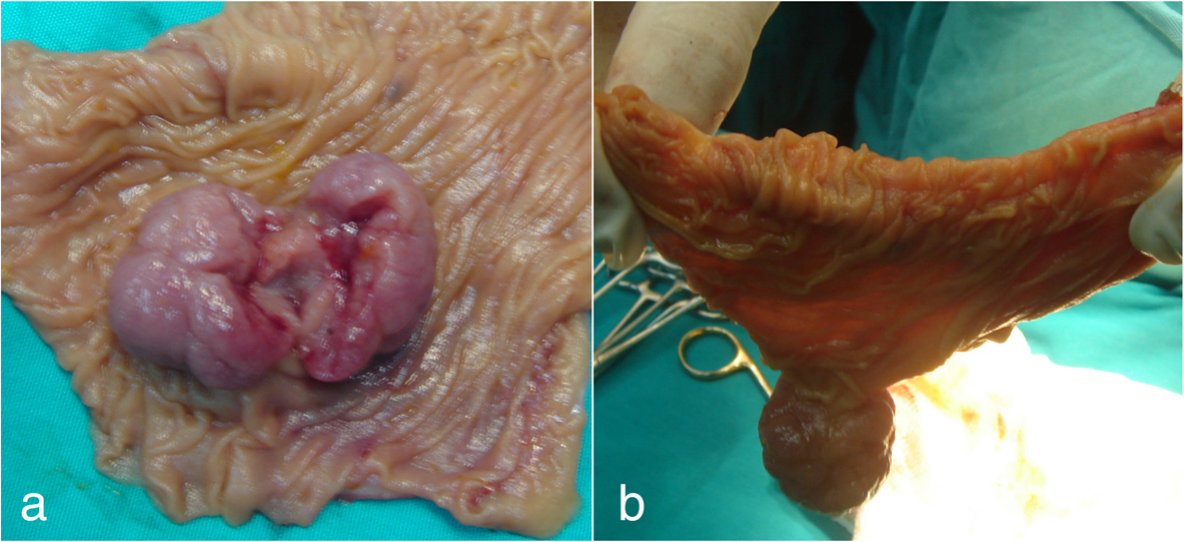
Post-operative macroscopic view of the surgical specimen (**a**), with a solitary broad-based polyp about 3cm in diameter (**b**).

Macroscopically, the excised polyp measured about 3cm (Figure 5) and was sent for pathological examination.

**Figure 6:**
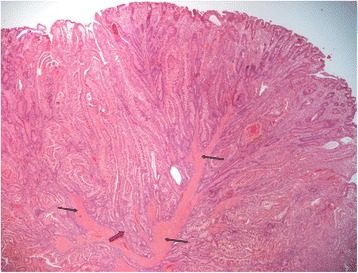
Hematoxylin and eosin–stained section of the Peutz-Jeghers polyp. The broad bands of smooth muscle fibers (black arrows) are characteristically thicker in the center of the polyp. The dark pink arrow shows a typical area of intermixing of glands and smooth muscle fibers mimicking invasion.

The pathology report confirmed the polyp to be a hamartoma with characteristics of a PJP (Figure 6).

Post-operatively, the patient had an uneventful recovery, and she did not experience any complications. Her recovery was quick, and she was discharged on post-operative day 4. Three months later, an endoscopic examination of the upper and lower gastrointestinal system was performed without confirmation of any polyps.

## Discussion

Small bowel intussusceptions induced by polyps can be managed successfully with minimally invasive surgical approaches. Laparoscopy is one of these approaches that deals with the acute problem, is a safe and effective method for reducing an intestinal intussusception and may prevent adhesion formation. Laparoscopy allows adequate access to explore and treat small bowel polyps and avoid the classic laparotomy [2,8,9]. The ideal way to remove a pedunculated polyp acting as a lead point is endoscopically. When this is not possible, laparoscopy is a safe and effective method of surgical management with reduction of the intussusception and small bowel resection [4]. The treatment of obstruction in these patients is performed by removing the hamartomatous polyp(s). The rest of the intestine needs to be examined, and those polyps found should be removed. This can be done intra-operatively with laparoscopy-assisted enteroscopy and colonoscopy [4].

The laparoscopy-assisted double-balloon enteroscopy is a recently developed technique that can be used as both surveillance and a therapeutic tool for small bowel polyps in patients with hamartomatous polyps [5,9,10].

A combined endoscopic and laparoscopic approach can be used to treat proximal small bowel intussusception, which could possibly eliminate the need for laparotomy and reduce the post-operative complications associated with multiple reoperations in this patient population [8].

Attempts to further minimize trauma related to surgical procedures and improve their cosmetic effects, especially in young patients, resulted in modification of the laparoscopic technique to a surgical procedure with a single, small incision, most commonly in the umbilicus [11].

SILS or one-port umbilical surgery is a more recent technique in the field of laparoscopic surgery. The first SILS case was reported in 1997. Complex surgical procedures can be performed through a single 2cm incision by using flexible endoscopes and articulating instruments. All surgical instruments are placed through this small incision [12]. In SILS, the size of the ports and the number of trocars are decreased to reduce abdominal wall trauma. The advantages of minimally invasive surgery include less post-operative pain, faster recovery, decreased incisional morbidity and improved cosmetic outcomes [10,13,14]. The progress from four incisions to a single incision has consistently shown better outcomes in terms of post-operative pain and cosmetic results and may reduce the risk of trocar site–related complications such as incisional hernia or infections [12].

SILS seems also to be a very effective surgical technique specifically with regard to PJPs. PJPs are usually sited in the jejunum and ileum (90% of the cases), colon (42%), stomach (38%) and rectum (28%) [2,4,6]. They consist of benign hamartomas, and the incidence of neoplastic change is between 3% and 6% at the time of diagnosis, with no relationship between the size of the polyps and their neoplastic character [3,8]. A solitary PJP is a rare, usually incidental finding. It is unclear whether a solitary PJP is an incomplete form of PJS or a separate entity, as the other phenotypic characteristics are lacking [1].

The diagnosis of PJS should be considered in patients who present with a clinical picture of bowel obstruction and mucocutaneous hyperpigmentation [4,6]. A complication of PJS is often intussusception, which is less frequent in the colon than in the longer, more mobile small intestine [7,15]. This condition can often result in bowel obstruction. A major early symptom is intermittent, moderate to severe cramping and abdominal pain. Intussusception can cause a loop of bowel to become necrotic secondary to ischemia caused by compression to the arterial blood supply. This leads to perforation and sepsis. As a result, surgical treatment is absolutely necessary [6-8]. If the diagnosis is made pre-operatively on the basis of US, colonoscopy or double-contrast gastrointestinal studies followed by magnetic resonance imaging, optimal management should include laparoscopic treatment of the bowel obstruction and intra-operative enteroscopy [1,4]. Surgery or laparoscopy combined with intra-operative enteroscopy is recommended for removal of any symptomatic polyp of the small bowel or any polyp larger than 1.5cm in diameter [3].

Ideally, preservation of intestinal length is important in patients with PJS because recurrence is seen in up to 10% of cases, and multiple resections could lead to the short bowel syndrome. However, recurrence of intussusception due to polyps is common in PJS, and a combined approach of laparoscopy and endoscopy to reduce the need for multiple laparotomies may avoid untoward problems in the future [8,9].

Despite the fact that we describe above the PJS, we have to mention that our patient had only a solitary bowel polyp, which appeared to be a PJP based on the pathology report, and that we did not diagnose our patient with PJS. Our report aims to present the surgical management of the intussusception caused by this solitary PJP by SILS.

To our knowledge, there are at least six other published case reports regarding the laparoscopic management of bowel obstructions in PJS. We searched the medical literature in PubMed by using the keywords “PJS”, “solitary PJP”, “small-bowel intussusception”, “SILS”, and “laparoscopy.” We analyzed this database and found only three articles about small bowel intussusception induced by a solitary PJP treated with laparoscopic management, but we found no reported results about SILS. There are only three reports about laparoscopic treatment of multiple polyps (Table 1).

**Table 1:** **Literature review of single-incision laparoscopic surgery in the treatment of Peutz-Jeghers-type polyps**

**Authors [reference citation]**	**Case report title**	**Type of polyp**	**Date of publication**
Cunningham *et al*. [8]	The role of laparoscopy in the management of intussusception in the Peutz-Jeghers syndrome: case report and review of the literature	Solitary polyp	1998
Hasegawa *et al*. [2]	Laparoscopic treatment of intestinal intussusception in the Peutz-Jeghers syndrome: case report and review of the literature	Solitary polyp	2006
Retrosi *et al*. [1]	Solitary Peutz-Jeghers polyp in a paediatric patient	Solitary polyp	2010
Gonzalez and Clapp [4]	Laparoscopic management of small bowel intussusception in a 16-year-old with Peutz-Jeghers syndrome	Multiple polyps	2008
Thomson *et al*. [10]	Double balloon enteroscopy in children: diagnosis, treatment, and safety	Multiple polyps	2010
Ross *et al*. [5]	Laparoscopic-assisted double-balloon enteroscopy for small-bowel polyp surveillance and treatment in patients with Peutz-Jeghers syndrome	Multiple polyps	2006

## Conclusions

Surgical procedures have strongly evolved from laparotomy to laparoscopy because of the development of laparoscopic equipment and improvement in surgical skills. Nowadays, SILS is one of the most minimally invasive laparoscopic techniques and has a lot of benefits, even in rare pathogenic situations. We think that this effective surgical approach can be equally used for the treatment of non-acute and acute cases, such as of a bowel intussusception. SILS can be a suitable choice for the treatment of intestinal intussusception induced by a solitary hamartomatous polyp and offers the opportunity to retain the whole length of the bowel as well as the benefits of its minimally invasive nature.

## Consent

Written informed consent was obtained from the patient for publication of this case report and accompanying images. A copy of the written consent is available for review by the Editor-in-Chief of this journal.

## Abbreviations

CT: Computed tomography
PJP: Peutz-Jeghers polyp
PJS: Peutz-Jeghers syndrome
SILS: Single-incision laparoscopic surgery
US: Ultrasound
